# Morpho-anatomical, and chemical characterization of some calcareous Mediterranean red algae species

**DOI:** 10.1186/s40529-023-00373-0

**Published:** 2023-04-18

**Authors:** Mona M. Ismail, Gehan A. Ismail, Mostafa E. Elshobary

**Affiliations:** 1 National Institute of Oceanography and Fisheries, NIOF, Cairo, Egypt; 2 Department of Botany, Faculty of Science, Tanta University, Tanta, 31527 Egypt

**Keywords:** Proximate contents, *Corallina officinalis*, *Jania rubens*, 18S rRNA gene, Physiochemical parameters

## Abstract

Climatic changes are anticipated to have a detrimental effect on calcifying marine species. Calcareous red algae may be especially vulnerable to seasonal variations since they are common and essential biologically, but there is little research on the morpho-anatomical, and chemical characterization of such species. This study conducted the seasonal investigation of the three dominant Mediterranean calcified red algae. Morphological and 18S rRNA analysis confirmed the identification of collected species as *Corallina officinalis, Jania rubens,* and *Amphiroa rigida*. In general, *C. officinalis* was represented in the four seasons and flourishing maximum in autumn (70% of total species individuals). While *J. rubens* species was represented in winter, autumn, and spring and completely absent in summer. *A. rigida* was abundant only in the summer season by 40%. A full morphological and anatomical description of these species were examined, and their chemical compositions (carbohydrate, protein, lipid, pigments, and elements content) were assessed in different seasons, where carbohydrates were the dominant accumulates followed by proteins and lipids. Pearson correlation analysis confirmed a positive correlation between salinity level and nitrogenous nutrients of the seawater with the pigment contents (phycobiliproteins, carotenoids, and chlorophyll a) of the studied seaweeds. The results proved that calcified red algae were able to deposit a mixture of calcium carbonates such as calcite, vaterite, calcium oxalate, calcite-III I calcium carbonate, and aragonite in variable forms depending on the species.

**Supplementary Information:**

The online version contains supplementary material available at 10.1186/s40529-023-00373-0.

## Highlights

- The abundance and diversity of the Rhodophyta species are threatened by global climate changes, rising sea levels, and stronger tidal waves.
- Shoreline hardening had direct impacts on the abundance and distribution of native species.
- Calcified species can be used as biomarker species for assessing climate changes.
- The morphological and 18S rRNA analysis confirmed the identified species.
- Only three species of red calcified algae viz. Corallina officinalis, Jania rubens, and Amphiroa rigida were recorded during seasons of autumn 2019−summer 2020.

**Supplementary Information:**

The online version contains supplementary material available at 10.1186/s40529-023-00373-0.

## Introduction

The Mediterranean Sea is the world's biggest (2,9 × 10^6^ km^2^) and deepest enclosed sea (average 1460 m, maximum 5267 m) (Coll et al. 2010). Even though it accounts for just 0.82% of the world’s surface area and 0.32% of its volume (Bianchi and Morri 2000), it will be one of the places most impacted by the continued warming trend and by an increase in severe conditions related to climate change, such as rising sea levels, increasing water temperatures, pH value, nutrients content loss of biodiversity, changes in species distribution, and an increase in extreme weather events such as storms and heatwaves (Galli et al. 2017). These changes may also lead to alterations in the ocean currents and circulation patterns, further impacting the ecosystem and the climate of the surrounding regions. Climate change is projected to have a significant influence on Mediterranean marine biota because it will interact with anthropogenic disturbances occurring at small scales (e.g., chemical pollution, eutrophication, increase in sediment load, and habitat degradation caused by trawling). Humans have used Mediterranean coastal habitats for millennia, altering them in a variety of ways. Nowadays, the Mediterranean coasts are densely urbanized and have a dense population; the effects of human activity are proportionately larger in the Mediterranean than in any other sea in the world (Coll et al. 2010). The primary causes of change include habitat destruction, deterioration and pollution, overexploitation of maritime resources, and alien species invaders, which will overlap and interact with climate-related changes in the next decades. Generaly, the Easter Harbor, Alexandria, Egypt is marked by abrupt temporal variations of multitudes of many distinct environmental variables, and experiences a high degree of pressure from both natural and anthropogenic activities (El-Dahhar et al. 2021).

Rhodophyta is the most diverse macroalgal group compared to Chlorophyta and Phaeophyta. Among Rhodophyta species, calcareous red species have been present in the Mediterranean algal community for about 140 million years (Chatalov et al. 2015) and are common on contemporary Mediterranean rocky coasts. These calcareous species are a multinational assemblage that are capable of precipitating calcium carbonate (CaCO_3_) within their vegetative cell walls (Walker et al. 2009). The carbonate skeleton of the calcareous species is crucial for their ecological existence and distribution, as it provides a shield defense against herbivores and resistance to water currents (Vásquez-Elizondo and Enríquez 2017). Also, the carbonate skeleton plays a master role in carbonate (CO_3_^2−^) productions for many marine carbonate biota such as coral reef communities. In addition, they are used as marine climate proxies via calibration of skeletal valorization morphology and geochemistry to environmental conditions (McCoy and Kamenos 2015). Besides, they are broadly utilized in palaeoecological studies archives (Jørgensbye and Halfar 2017).

The order Corallinales is considered to be the third most species-rich order within the Rhodophyta after the orders Ceramiales and Gigartinales (Brodie and Zuccarello 2007). The members of this order are distributed in marine wave−exposed littoral and sub−littoral habitats around the world and comprise both crustose and geniculate (articulated) forms (Walker et al. 2009). Corallinales members have been documented in the Mediterranean area fossil record back to the Oligocene era about 140 million years ago (Bianchi and Morri 2000). In the Mediterranean Sea, corallines algae cover a high spatial and bathymetric range that is evaluated to exceed 2700 km^2^ on the surface. Up till now, the classification of Mediterranean corallinaceae rely almost totally on gross morphology and morpho−anatomical data (observed by light, scanning, and/or electron microscopy) as well as the recent studies integrating molecular investigations (Kogame et al. 2017; Pardo et al. 2015). At present, 60 species of calcareous red algae are recorded for the Mediterranean coast; nevertheless, this estimated number of species is believed to be much lower than the expected real number of species as reported by Rindi et al. (2019). Among them, the genus *Lithophyllum* is the most abundant as represented by 16 species, followed by *Mesophyllum* (6 species), and *Amphiroa*, *Jania*, and *Lithothamnion* genera (5 species each) (Cormaci et al. 2017; Rindi et al. 2019). Rapid changes in seawater chemistry, especially lower pH, and changes in carbonate levels in surface waters, have been demonstrated to have a variety of negative effects on marine calcifying organisms (Smith et al. 2012; Cornwall et al. 2017; Barakat et al. 2021; El-Sayed et al. 2022). The calcium carbonate polymorphs will be influenced by the morphological identification of such species. It is well known that species identifications can be problematic, especially in calcareous red seaweeds (Brakel et al. 2021). In several studies of crustose coralline algae, numerous examples of misidentification were found (Hind et al. 2014; Cornwall et al. 2019). DNA Barcoding has become a common approach in calcareous red algal studies (Calderon et al. 2021). Over the years, the scientific community has conducted extensive research on coralline-dominated communities, exploring their assembly, dynamic distribution, and ecological significance. These studies have highlighted the impact of human activities on these communities, including the effects of climate change, ocean acidification, and overfishing (Hind and Saunders 2013; Cornwall et al. 2019), but still, the biology of Mediterranean corallines is limitedly recognized. Few studies investigated the influence of climate changes on Mediterranean corallines (Martin et al. 2013; Cornwall et al. 2017), and their acclimatizing or sensitivity response to higher temperatures, atmospheric CO_2_ concentrations and acidification of water (Marchini et al. 2019; El-Sayed et al. 2022). However, the field is still lacking a wider taxonomic record to coralline species along the whole Mediterranean areas from west to east (Cormaci et al. 2017). According to Rindi et al. (2019) and Peña et al. (2021), future studies on Mediterranean corallines should combine different fields like phylogeography, genomics, transcriptomics, and related microbiomes in which the knowledge of coralline algae is deficient. Although the Mediterranean coast is one of the main climate change hotspots, there is little information regarding the coralline algae species morpho-anatomy characterization in this area. In light of the above facts, this study is designed to describe the morpho-anatomy of the dominant calcareous (Rhodophyta) species found on Mediterranean coast, Alexandria, Egypt, and to infer their phylogenetic position at the species level using 18 s rRNA marker. Additionally, the phytochemical, proximate constituents, twenty elements and calcium carbonate content of these species were also investigated estimated in relation to the estimated physiochemical characteristics of their habitats and the season of collection.

## Materials and methods

### Chemicals

All the chemicals used were of analytical ranking and purchased from Sigma−Aldrich Company, analytical grade products, and used according to the manufacturer's guidance**.**

### Collection, processing, and authentication of the calcareous samples

Fresh calcareous red algal species were handpicked twice seasonally (viz*,* from autumn 2019 to summer 2020) at a depth of 0.5−1.5 m from the water of Eastern Harbor rocky shore, Alexandria city, Egypt (Longitudes 29.88º−29.90º E and latitudes 31.20º−31.22º N). Seaweed assemblages were sampled seasonally by a square of 4 m^2^ (2 × 2 m) at 10 m with a square metal frame (Dadolahi and Savari, 2005), and the regional abundance distribution (% of total species individuals) of different algal species was estimated.

The collected samples were preserved in plastic bags containing seawater to prevent evaporation. The samples were immediately transferred to the National Institute of Oceanography and Fisheries, Alexandria (Taxonomy and biodiversity of aquatic biota laboratory). The gathered seaweeds were washed using tap water followed by distilled water to remove all the epiphytic and foreign particles such as sand, rocks, or plastics wastes. After washing, ten thalli of each species (about 30–50 g fresh weight) were preserved in 5% formalin in seawater for taxonomic identification. The other portion (800–1000 g fresh weight) was lifted to shade dry at room temperature (27 ± 2 °C) for 48 h. After complete dryness in over at 45ºC for overnight, seaweed samples were finely powdered using an electric blender to pass through No. 40 mesh-size particles sieving instrument (around 0.42 mm in diameter). Each sample powder was stored in a separate dark vial for further investigation. For the phylogenetic analysis, 1 g fresh weight of each thallus was dried with silica gel desiccant (Cassano et al. 2012). The collected species were identified according to Aleem (1993) and Jha et al. (2009). The species names were authenticated according to Guiry and Guiry (2021).

### Physiochemical criteria of the seawater of the collection sites

The physical parameters (temperature, salinity, and pH) of surface seawater were recorded in situ, by multi−parameter instruments (HANA, Model HI 9828) at the error range of T ± 0.2 °C, the salinity of Sal. ± 0.01 PSU, and of pH ± 0.1. The chemical parameters of water samples “dissolved oxygen (DO), nitrates (NO_3_), nitrites (NO_2_), ammonia (NH_4_), and phosphates (PO_4_)” were performed according to American Public Health Association (APHA 1998).

### Macroscopic analysis of the collected calcareous samples

The morphological study was carried out on the collection day. The macroscopical examinations involved the main external features of length (cm), and type of branching (mono, di, tri….) for different thallus parts. Color assessment was conducted by visually observing the samples under natural light.

### Microscopic analysis of the collected calcareous samples

Microscopic evaluation was conducted for the whole thallus of the collected samples (three or five filamentous from each thallus). Transverse and longitudinal hand−sections were prepared using a stainless−steel razor blade and then stained with aqueous aniline blue solution (0.5%), acidified with 1N HCl (Tsuda and Abbott 1985). Then they were examined under a compound microscope (model BEL Bio-1-T, with total magnification up to 1000 x, and photographed with a Nikon digital camera (model D5000).

### Molecular identification of the collected calcareous samples using 18S rRNA gene

Three samples (1 g dry weight) of each species were subjected to the same DNA extraction protocol, and the resulting DNA was used for downstream molecular analyses. Total genomic DNA was extracted according to the manufacture protocol of Gene JET Genomic DNA Purification Kit Thermo DNA Purification Kit (K0721/Thermo Scientific). PCR amplification of partial 18S rRNA gene sequences was carried out using the forward primer CDMF (5ʹ − GTCAGAGGTGAAATTCTTGGATTTA − 3ʹ) and reverse primer CDMR (5ʹ − AAGGGCAGGGACGTAATCAACG − 3ʹ) (Gross et al. 2001). Amplification protocol was carried out according to Elshobary et al. (2015), using thermal cycler conditions (Applied Biosystems 2720, Foster City, CA, USA) as follows: initial 94 °C for 5 min, followed by 30 cycles of denaturing at 94 °C for 30 Sec, annealing at 60ºC for 1 min and elongation at 74 °C for 1 min, then a final extension step at 74 °C for 10 min. Agarose gel (1%) electrophoresis was used to detect the amplified PCR products, which were recovered and purified using Promega’s Wizard SV Gel and PCR clean−up system (Promega Corp., Madison, Wisconsin, USA). The Gel documentation system (Geldoc−it, UVP, England), was applied for data analysis using Totallab analysis software, ww.totallab.com, (Ver.1.0.1). Both strands were sequenced with Big−Dye 3.1 as described by Elshobary et al. (2015). Sequence analysis was employed using the ABI PRISM® 3100 Genetic Analyzer (Micron−Corp. Korea). Aligned sequences were analyzed by the NCBI website (https://blast.ncbi.nlm.nih.gov) using nBLAST to confirm their identity. The sequences have been aligned using Clustal W with the default parameters (MEGA X software) (Kumar et al 2018). Using MEGA X software, a dendrogram was created using maximum likelihood (ML), Tamura−Nei model (Tamura and Nei 1993), and maximum parsimony (MP) utilizing the option for tree bisection and reconnection branch embedded in MEGA X (Nei and Kumar, 2000). 1000 bootstrap resampling were used for node support (Felsenstein 1985). Bootstrap was performed in MEGA X for ML and MP, and values of more than 70 are given in the trees.

### Qualitative phytochemical analysis

The qualitative phytochemical screening of the methanolic extract of each seaweed sample was essentially carried out to evaluate their various phytoconstituents such as steroids, tannin and glycosides, etc. (Raman 2006).

### Proximate composition and pigments content

Total carbohydrate, proteins, lipids, and photosynthetic pigments contents were estimated in the investigated seaweeds as per standard methods. Total carbohydrates were evaluated using the phenol−sulphuric acid method (Dubois et al. 1956), after extraction of the powdered samples with HCl (2.5 N) for 3 h at 100 °C. For total proteins, dry seaweed materials were, firstly, extracted overnight into Tris HCL buffer (0.1 M pH 7.5) while stirring at 4 °C. After centrifugation, the proteins in the supernatant were measured spectrophotometrically at 750 nm (Lowry et al. 1951) using UV − spectrophotometer (Spectronic 20 colorimeter Stat Lab Version SZSL 0198, USA). The total lipids content of seaweed samples was estimated gravimetricallyy according to Daneshvar et al. (2018) method after extraction with (2:1) chloroform: methanol mixture solution. All the proximate analysis results were expressed as a percentage (%) of the sample dry weight.

For the pigments content estimation, 500 mg of each algal sample were extracted twice in 10 ml of 80% acetone (Arnon 1949). The absorbance of the collected supernatants was measured at 645 nm and 663 nm using UV−spectrophotometer. In addition, the carotenoid content of the seaweed samples was estimated spectrophotometrically at 480 nm using the same extract (Kirk and Allen 1965). The pigments content were calculated using the following Eqs. (1–3) and were finally stated as mg/g fresh weight of each sample.

1 $${\text{Chloraphyll}}\,{\text{'a'}} = \frac{{[12.7({\text{A}}_{663} )\, - \,2.69({\text{A}}_{645} )]\,\, \times \,{\text{vol}}{.}\,{\text{of}}\,{\text{extraction}}}}{{{\text{Weight}}\,{\text{of}}\,{\text{sample}}}}$$

2 $$Total\,Chl\, = \,\frac{{[20.2(A_{645} )\, - \,2.69(A_{645} )]\, \times \,{\text{vol}}{.}\,{\text{of}}\,{\text{extraction}}}}{{{\text{Weight}}\,{\text{of}}\,{\text{sample}}}}$$

3 $$Carotenoids\, = \,\frac{{4\, \times (A_{663} )\,{\text{vol}}{.}\,{\text{of}}\,{\text{extraction}}}}{{{\text{Weight}}\,{\text{of}}\,{\text{sample}}}}$$

For the estimation of phycobiliproteins (Phycocyanin (PC), Allophycocyanin (APC), and Phycoerythrin (PE) pigment, the seaweed samples were extracted in phosphate buffer (0.1 M) according to Padgett and Krogman (1987), and calculated as mg/g fresh weight using the following Eqs. (4–6):

4 $${\text{PC}}\, = \,\frac{{({\text{A}}_{{{615}}} ) \times (0.475 \times {\text{A}}_{652} )}}{5.34}$$

5 $${\text{APC}}\,{ = }\,\frac{{({\text{A}}_{652} ) \times (0.208 \times {\text{A}}_{615} )}}{5.09}$$

6 $${\text{PE}}\,{ = }\,\frac{{({\text{A}}562)\, - \,(2.41 \times {\text{PC)}}\, - \,(0.849\, \times \,{\text{APC)}}}}{9.62}$$

where: A_562_, A_615,_ and A_652_ = absorbance values at 562, 615, and 652 nm, respectively.

### ***Estimation of CaCO***_***3***_*** content***

The accumulation of CaCO_3_ in each sample was estimated gravimetrically, according to Sinutok (2013). A known weight of dry algal samples were hydrolyzed in 5% HCl for 1 h, and the dry weight loss after CaCO_3_ dissolution was determined and expressed as mg/g dry weight.

#### X-Ray diffraction analysis

The crystalline composition and structure of the three red algal samples were characterized by X-ray diffraction analysis (X-ray Powder Diffraction, XRD-D2 phaser) with CuKα radiation, λ = 1.54184 Aº at 30 kV and 10 mA. The diffraction pattern was collected at a scanning rate of 0.02 degrees per s and 2θ range of 10° to 100°.

#### Analysis of elements’ content

Ten element contents viz*.,* calcium (Ca), magnesium (Mg), cadmium (Cd), chrome (Cr), copper (Cu), iron (Fe), manganese (Mn), nickel (Ni), lead (Pb), and zinc (Zn) were assessed in the collected species. The samples were first hydrolyzed in a mixture of HNO_3_ and HClO_4_ (2:1 V/V) for 12 h at room temperature and heated at 100 °C for 2 h to decolorize the samples. After cooling, solutions were made up to 20 ml with deionized H_2_O, and then measured using atomic absorption spectrophotometer (PFP-7, Jenway, Japan) and expressed as μg/g and/or mg/g dry weight.

### Statistical analysis

All the measures were carried out in triplicates as a minimum replica. The results were expressed as mean value ± standard deviation (SD). For comparing means between parameters, one way ANOVA was established using SPSS base 15.0 software (Chicago, USA: Users guide SPSS Inc., 2006) at *P* < 0.05 level of significance. Pearson’s correlation analysis (at α ≤ 0.2) between the environmental physicochemical parameters and the biochemical compositions of the studied calcified species was performed using SPSS software to determine their relationship.

## Results and discussion

### Seasonal variation in the abundance of the collected calcareous species

**Fig. 1:**
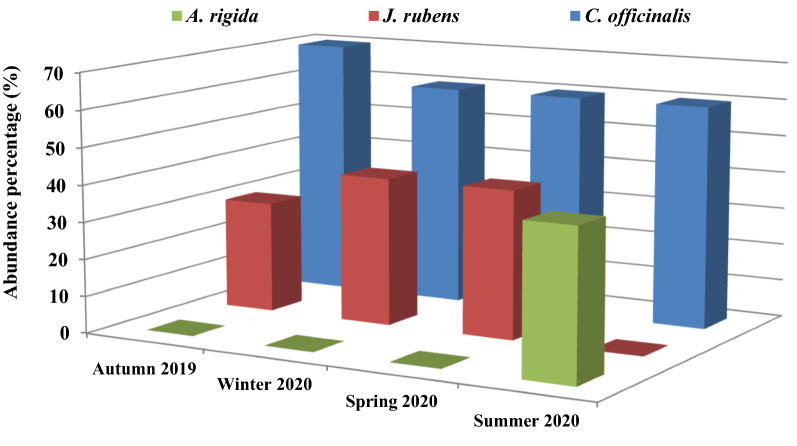
Seasonal abundance distribution (% of total species individuals) of the collected calcified species

There was a varied seasonal abundance of the collected calcified red species, especially during autumn, spring and summer, as shown in Fig. 1. A low diversity was noticed for the collected samples, and their abundance greatly fluctuated during different studied seasons. Only three species were collected during the study period, identified morphologically according to the previously mentioned taxonomical reference guides, and then verified with the Algae Base website (Guiry and Guiry 2021) as *Amphiroa rigida* Lamouroux*, Corallina officinalis* Linnaeus*,* and *Jania rubens* (Linnaeus) Lamouroux. *C. officinalis* was the dominant species in all seasons with a maximum abundance of 70% in autumn while represented by 60% in the other seasons (Fig. 1). This could be related to its physiological tolerance to a broad range of temperature, DO, NO_2_, and NH_4_ concentrations during the study period (Table 1). The same conclusion was reported by Kim et al. (2018) that *C. officinalis* was tolerant to varied environmental conditions of light, desiccation, and grazing. In comparison, autumn showed the lowest abundance percentage of *J. rubens,* recording 30% of the total collected species. This species was represented equally with 40% of the total collected species in winter and spring seasons, while completely absent in summer. Besides, *A. rigida* was only detected in the summer season with 40% of the total species individuals. This may be due to the significant (at *p* ≤ 0.05) temperature and NH_4_ concentration increase in combination with the significant decrease in pH, DO, and PO_4_ concentrations (Table 1). The correlation results listed in Table 4 confirmed this relation between the species abundance and pH, DO, and NH_4_ concentrations. In this connection, Anggadiredja (2017) informed that biodiversity and developments of seaweed species are regulated by physicochemical parameters such as temperature, light and salinity, and interaction with other biota. Calcified red species have traditionally flourished in warm water habitats (Jørgensbye and Halfar 2017), which was in agreement with the obtained results. Also, Osman et al. (2020) demonstrated similar results that *C. officinalis* was recorded in all seasons of 2019–2020 from Abu Qir Bay of the Mediterranean Sea of Egypt, while *J. rubens* was not represented in summer. Another parameter that was not assested in this study but might vitaly affected the abundance and biodiversity of the three studied species was the increased undisciplined fishing, shipping and shoreline hardening practices. These anthropogenic activities were manifested on enlarged scale in the shoreline area during the study period. According to Gittman et al. (2016), engineered shore structures have decreased the biodiversity by 30% and the abundance of aquatic biota by 45% more than natural shorelines, implying that these constructions are having a negative impact on coastal ecosystems.

**Table 1:** Physiochemical criteria per season of the collected seawater samples from Eastern harbor^a^

Season	Temperature (°C)	Salinity (PSU)	pH	DO(mg/L)	NO_3_(µM)	NO_2_(µM)	NH_4_(µM)	PO_4_(µM)
Autumn 2019	21.08^c^ ± 1.34	37.46^c^ ± 0.34	7.983^a^ ± 0.21	6.00^a^ ± 0.12	1.801^b^ ± 0.11	6.577^c^ ± 0.68	2.678^b^ ± 0.32	2.123^c^ ± 0.11
Winter 2019	13.37^a^ ± 2.45	35.74^a^ ± 0.11	8.505^b^ ± 0.11	10.50^c^ ± 0.21	1.786^b^ ± 0.03	6.700^c^ ± 0.81	4.025^c^ ± 1.04	2.155^c^ ± 0.06
Spring 2020	18.72^b^ ± 1.98	36.88^b^ ± 0.21	8.465^b^ ± 0.13	8.86^b^ ± 0.11	1.946^c^ ± 0.004	2.374^b^ ± 0.26	1.935^a^ ± 0.10	1.938^b^ ± 0.24
Summer 2020	29.23^d^ ± 1.23	37.76^c^ ± 0.54	8.093^a^ ± 0.22	6.07^a^ ± 0.14	1.188^a^ ± 0.02	1.330^a^ ± 0.03	3.750^c^ ± 0.63	1.183^a^ ± 0.01

^a^Values are means of three replicates ± SD**.** Different superscript letters in the same column means significant difference (at *p* ≤ 0.05) between seasons for the same parameter

### Physicochemical parameters analyses of the collection sites

As shown in Table 1, fluctuated temperature values were noticed during seasons. Winter recorded the lowest temperature (13.37 ± 1.34 °C), while summer temperature was the highest (29.32 ± 1.23 °C). However, these values remained in the optimal range (16 °C- 31 °C) commonly known for the Mediterranean shores of the collection sites (El-Dahaar et al. 2021; Dango et al. 2015). Salinity values followed a pattern similar to temperature. The level of salinity significantly (at *p* ≤ 0.05) decreased in winter (35.74 PSU), and spring (36.88 PSU) compared to the autumn (37.46 PSU) and summer (37.76 PSU) seasons of the collection year (2019−2020). This irregular temporal trend of salinity in Eastern Harbor may be related to intensive dilution via additional amount of municipal wastewater from the kayet Bey sewer, located at its western vicinity, as well as from Mex Bay in the west which reduces the surface and bottom salinity particularly during spring (Labib et al. 2023). In addition to the increase evaporation of the water by high temperature during summer. In this regard, a positive relationship between water temperature and salinity (r = 0.91) has been reported by Xiong et al. (2013). A similar trend was recorded in a recent studies by Labib et al. (2023) in Eastern Harbor, and Osman et al. (2020) in Abu Qir Bay of the Mediterranean Sea of Egypt. Meanwhile, the pH value was stable in the weak alkaline range from a minimum value 7.98 in autumn to a maximum value of 8.51, in winter, indicating no ambient acidification effects. The DO values were the maximum in winter (10.5 mg/L) followed by spring (8.85 mg/L), although significantly (at *p* ≤ 0.05) it decreased to about 6 mg/L during autumn and summer of the study period. A strong negative correlation value (r =  − 0.85) was calculated between the DO levels and the temperature values in all seasons. These results were in conformity with many reports that the level of DO differs inversely with temperature and dissolved solids content of the seawater. In addition, the estimated values for nitrites (NO_2_) and phosphates (PO_4_) were the highest in autumn and winter seasons compared to summer and spring times, while NO_3_ and NH_4_ were tangled between seasons, which was reflected by a strong negative correlation between these nutrients concentration in the seawater and the temperature values (Table 4). These outcomes were similar to those obtained by (Osman et al. 2020).

### Seasonal variation in the morphological and anatomical features of the collected calcareous species

**Fig. 2:**
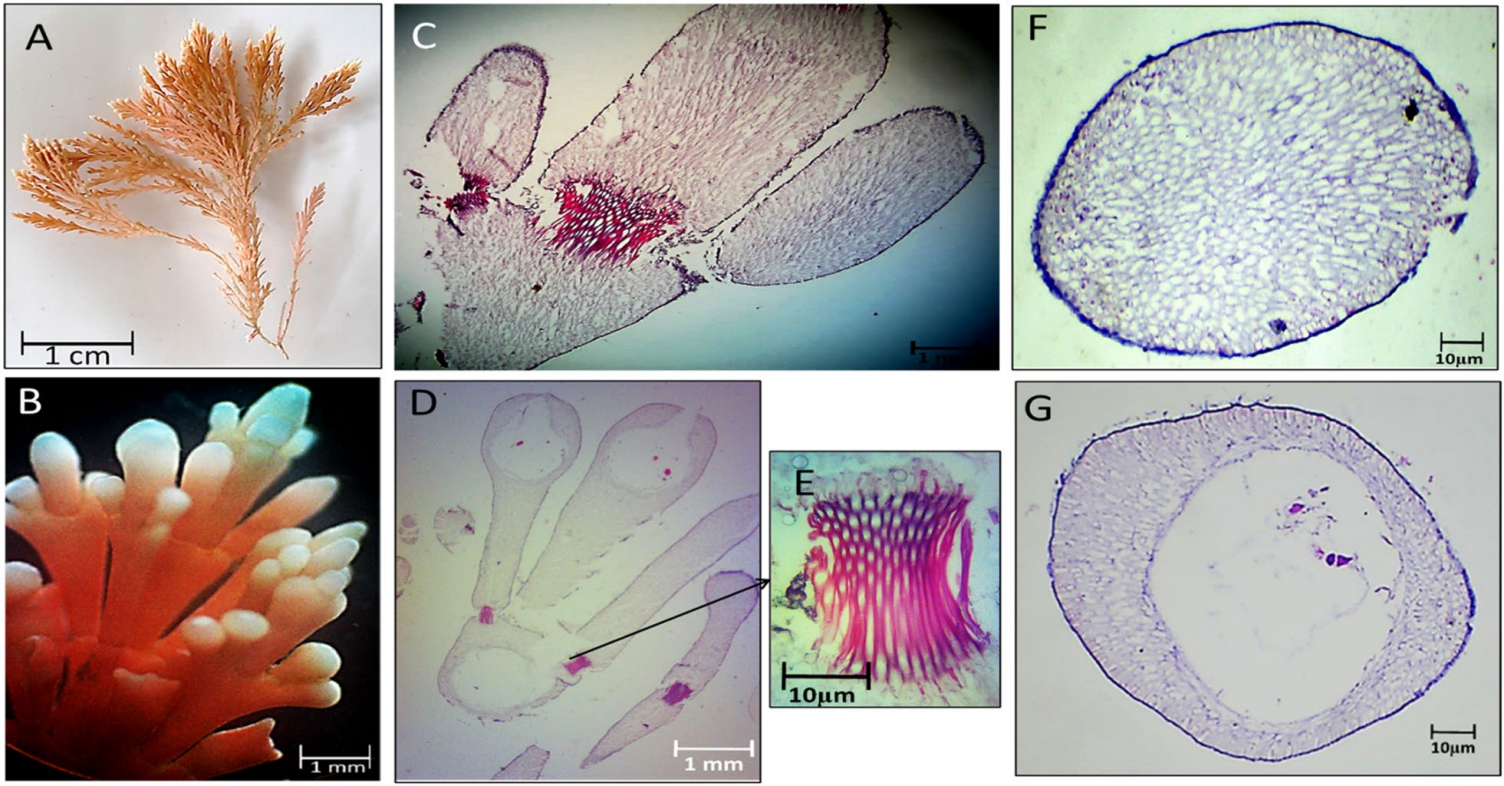
Morphological and anatomical description of *C. officinalis* thallus during autumn 2019. **A** Habit view of thallus, **B** Magnification of the thallus frond **C** L.S. of thallus showing genicula and intergenicula tiers. **D** L. S. through mature geniculum and intergenicula, **E** Mature geniculum with polygonal cells. **F** Cross-section through intergeniculum **G** Cross-section through tetrasporangial conceptacle

**Fig. 3:**
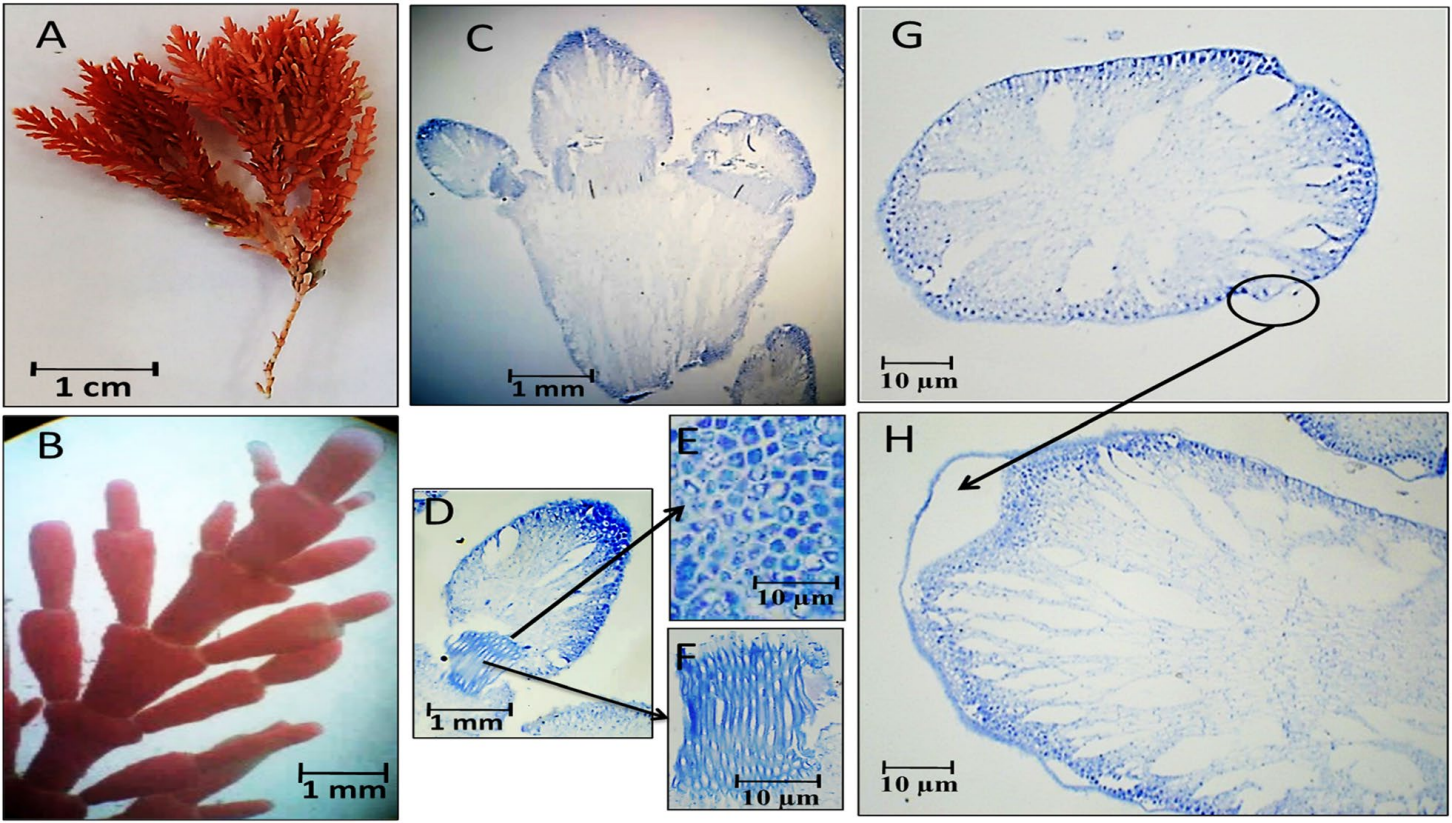
Morphological and anatomical description of *C. officinalis* thallus during winter 2019. **A** Habit view of thallus, **B** Magnification of the thallus frond, **C** L.S. in thallus showing genicula and intergenicula tiers, **D** L. S. through Magnification of mature geniculum and intergenicula, **E** Mature geniculum with polygonal cells, **F** Cross-section through intergeniculum, **G** and **H**Cross-section through intergeniculum

**Fig. 4:**
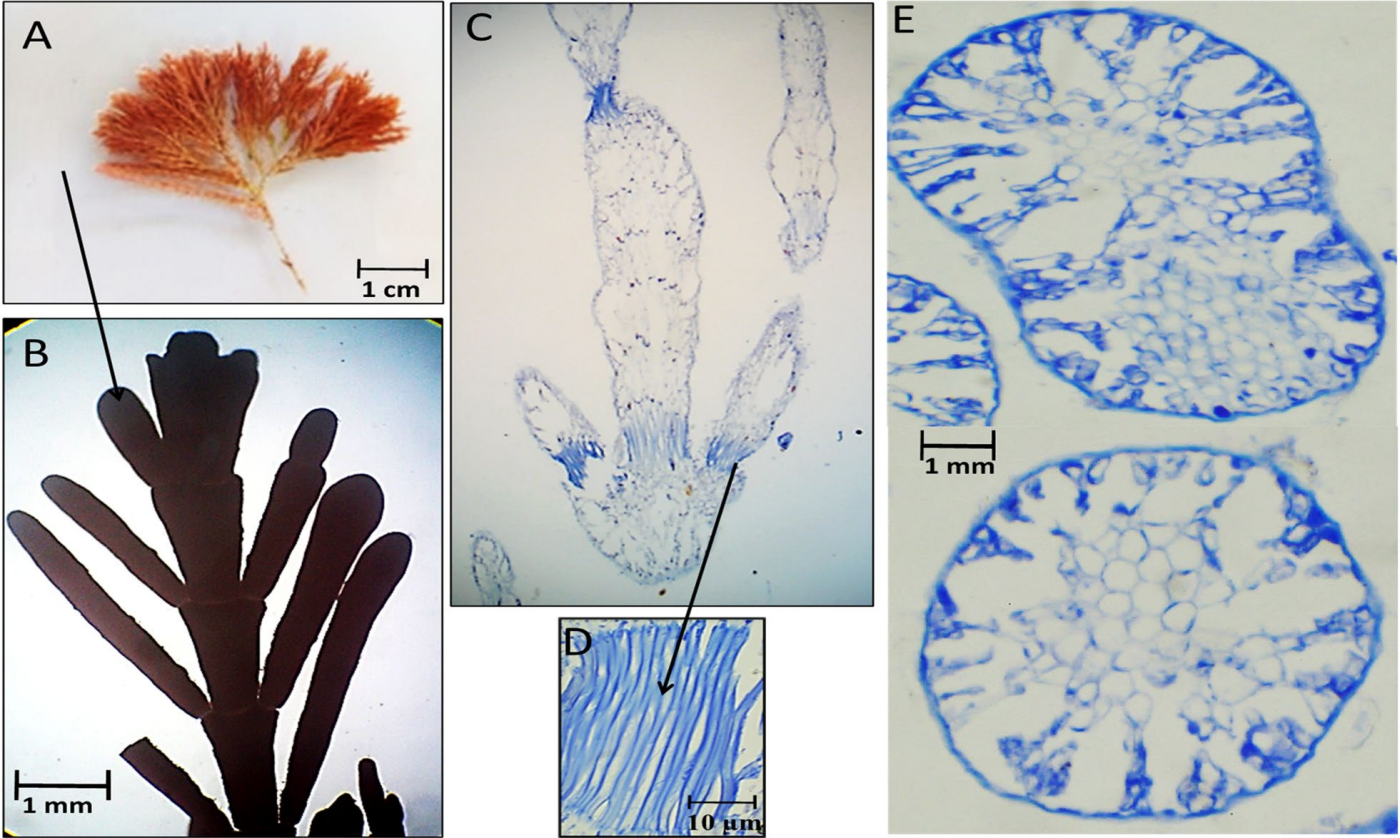
Morphological and anatomical description of *C. officinalis* thallus during spring 2020. **A** Habit view of thallus, **B** Magnification of the thallus frond, **C** L. S. in thallus showing genicula and intergenicula tiers, **D** Magnification of mature geniculum, **E)**Cross-section through intergeniculum

**Fig. 5:**
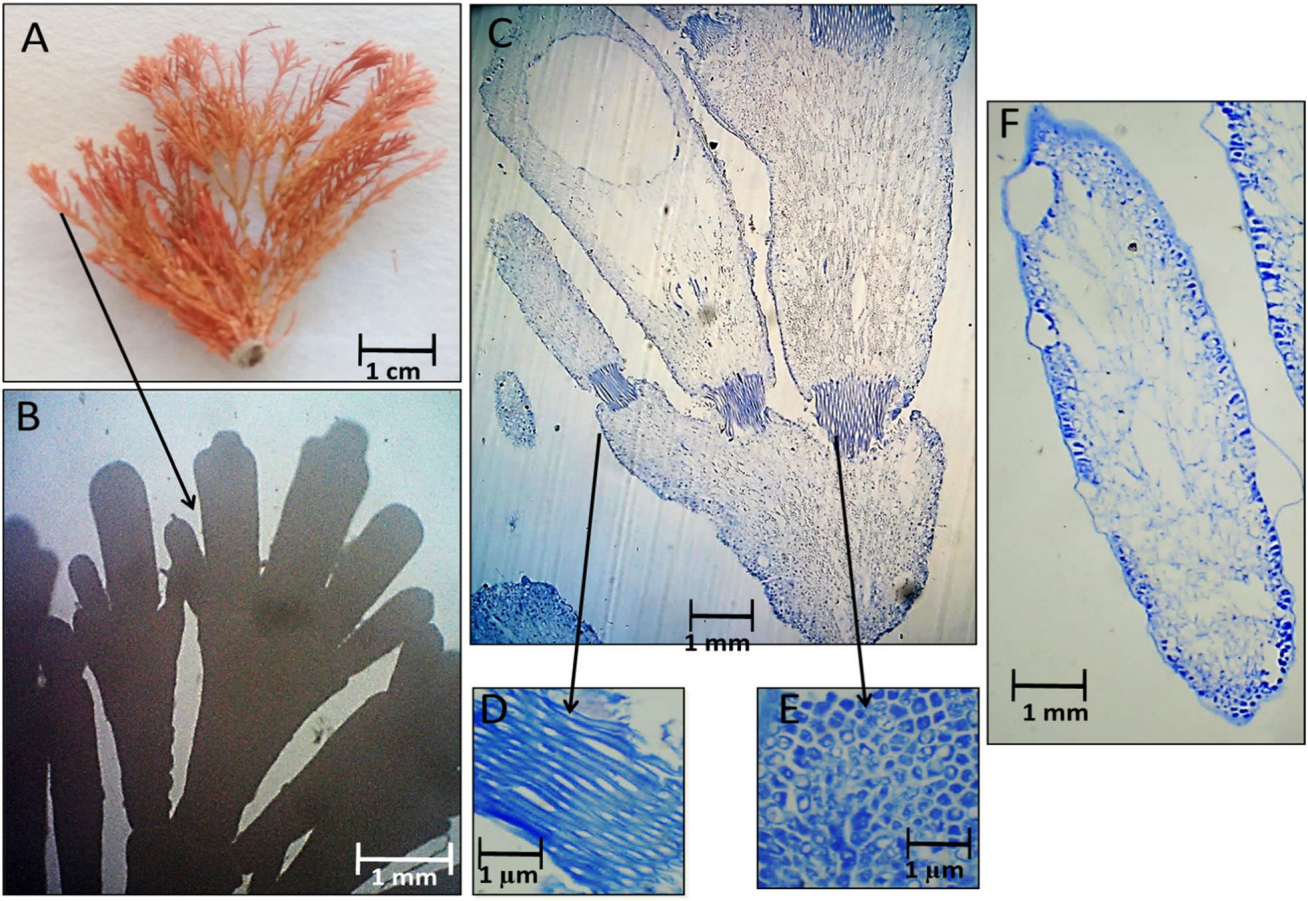
Morphological and anatomical description of *C. officinalis* thallus during summer 2020. **A** Habit view of thallus, **B** Magnification of the thallus frond, **C** L.S. in thallus showing genicula and intergenicula tiers, **D** and** E** Magnification of mature geniculum, **F** Cross-section through intergeniculum region

**Fig. 6:**
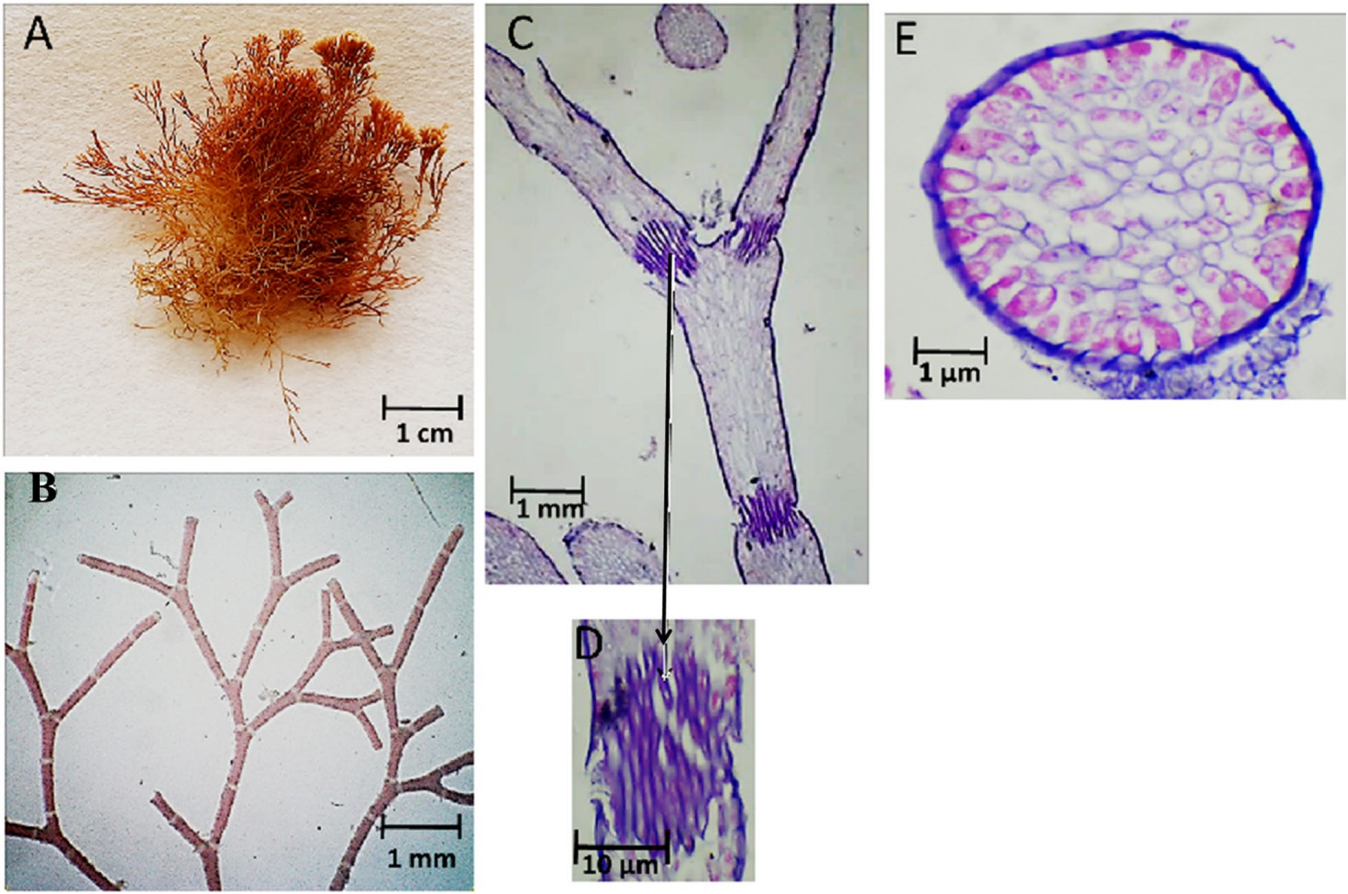
Morphological and anatomical description of *J. rubens* thallus during autumn 2019. **A** Habit view of thallus, **B** Magnification of the thallus frond, **C** L.S. in thallus showing genicula and intergenicula tiers, **D**) Magnification of mature geniculum, **E** and **E** Cross-section through intergeniculum region

**Fig. 7:**
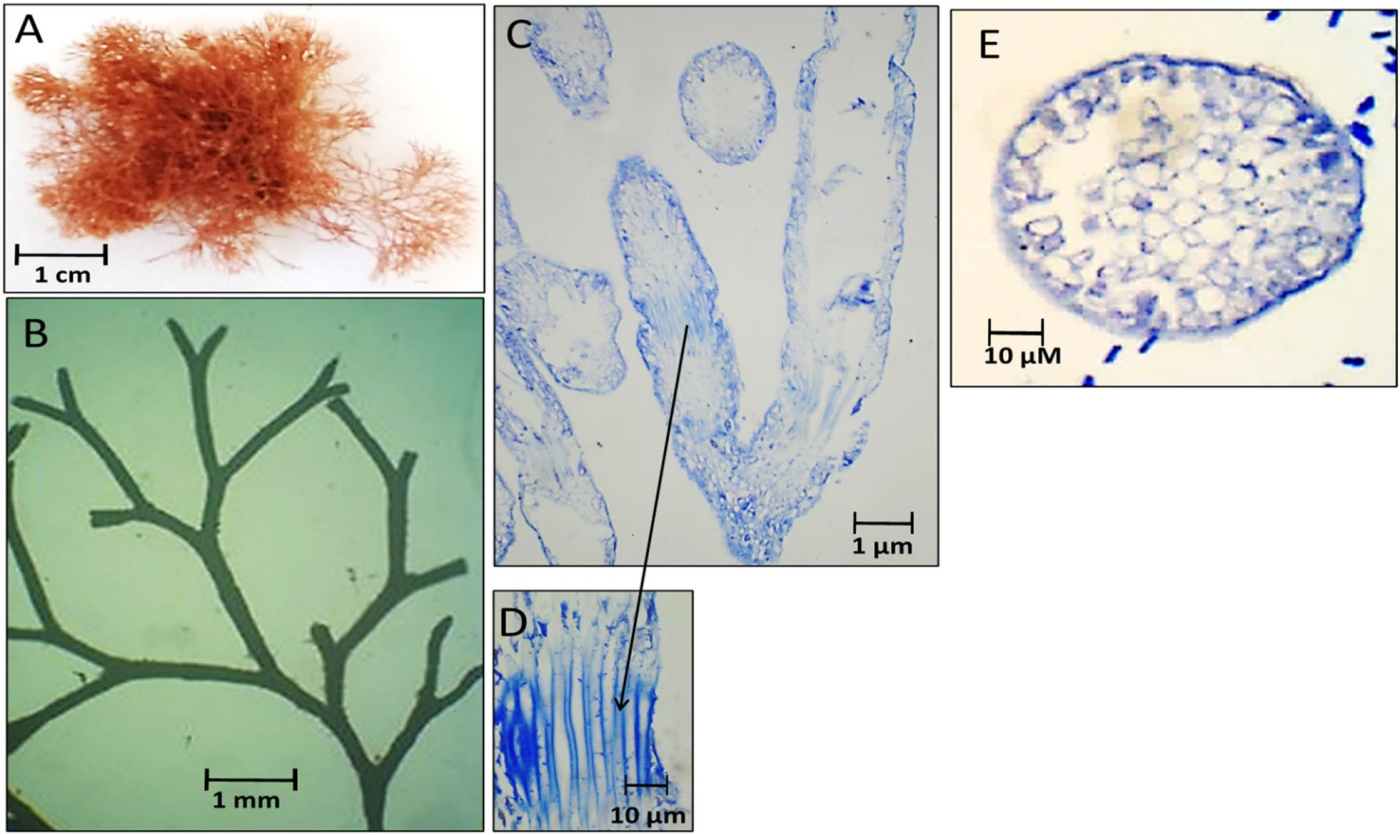
Morphological and anatomical description of *J. rubens* thallus during winter 2019. **A** Habit view of thallus, **B** Magnification of the thallus frond, **C** L.S. in thallus showing genicula and intergenicula tiers, **D** Magnification of mature geniculum, **E** Cross-section through intergeniculum region

**Fig. 8:**
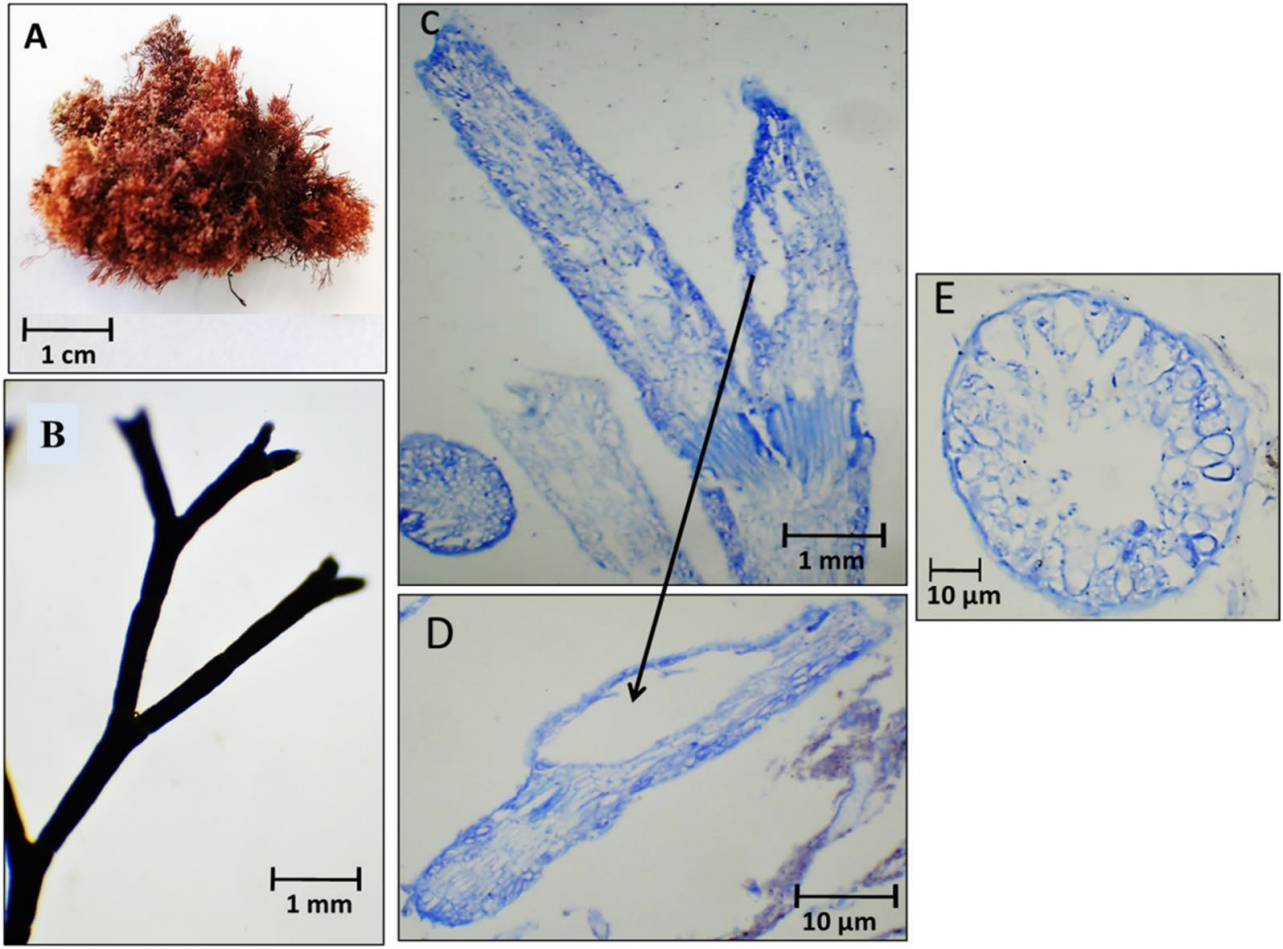
Morphological and anatomical description of *J. rubens* thallus during spring 2020. **A** Habit view of thallus, **B** Magnification of the thallus frond, **C** L. S. in thallus showing genicula and intergenicula tiers, **D** Magnification of mature empty tetrasporangia, **E** Cross-section through intergeniculum filament

**Fig. 9:**
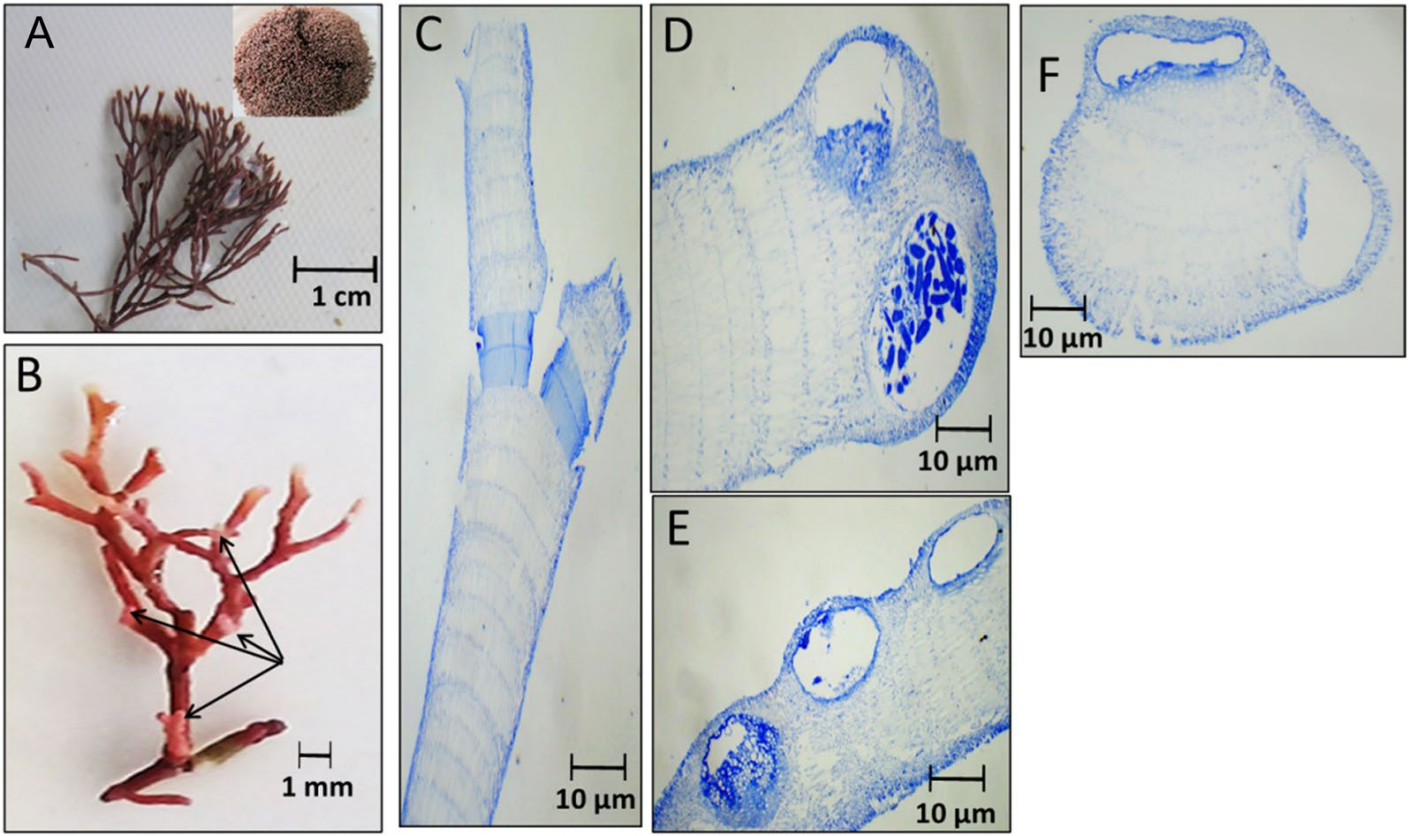
Morphological and anatomical description of *Amphiroa rigida* during summer 2020. **A** Habit view of thallus, **B** Magnification of the thallus frond, **C** L. S. in thallus showing genicula and intergenicula tiers, **D** Magnification of mature tetrasporangium at thallus tips, **E** Mature tetrasporangium at thallus peripherals of the intergeniculum, **F** Cross-section through intergeniculum filament

The changes in external appearance and anatomy, including the intergenicula, genicula, and reproductive structure of the collected red species were explained in the following figures during different seasons. Figures 2, 3, 4 and 5 illustrated *C. officinalis* thallus; and Figs. 6, 7, 8 and 9 illustrated *J. rubens* and *A. rigida* thalli.

During autumn 2019, the thallus length was up to 3–3.5 cm with a 1.5–2 cm lateral branch with red-pink color, and the erect stem appears segmented (Fig. 2A). The frond of the thallus is fragile and regularly trichotomy branched with a rounded tip with tiny gaps between lateral branchlets. Note that intergenicula were cylindrical, trapezoidal, and winged with white tips (Fig. 2B). The longitudinal section (L.S.) of geniculum and intergenicula tiers with three branches was shown in Fig. 2C, D. L.S. showed the mature geniculum (dark-red staining region) and intergenicula (lighter area) with tetrasporangial conceptacle. Mature geniculum composed of many polygonal cells (Fig. 2E). Cross-section through slightly compressed intergeniculum showed tiers of cortical cells, the epithelial concavities, which consist of the calcified proximal and lateral walls of the epithelial cells. They have a symmetrical-rounded outline (Fig. 2F). The cross-section through tetrasporangial conceptacle showed the same layers with a central cavity of the oospore (Fig. 2G).

As revealed by Fig. 3, *C. officinalis* thallus was red in color in winter season and up to 3.5–4 cm tall with lateral branches of 1.2–1.5 cm long (Fig. 3A). Intergenicula were long cylindrical with three symmetrical branches (Fig. 3A). The L. S. through thallus showed short geniculum and long intergeniculum tiers (Fig. 3C). The enlarged section through geniculum area showed core region of longitudinal medullary cells and peripheral region of irregular polygonal cells (Fig. 3D, E and F). The cross-section through intergeniculum region exposed loose cells with many intercellular spaces and calcified peripheral cell layers (Fig. 3G, H).

During spring 2020, *C. officinalis* thallus appeared purple-red in color, up to 2.0 cm tall and 0.7–1.0 cm long of lateral branches (Fig. 4A). The elongated apical intergenicula were flattened and trifurcate with obtuse apices and conspicuous gaps between lateral branchlets (Fig. 4B). The L. S. through the thallus showed transverse arrangement of intergeniculum cortical cells and the shortened geniculum area of filamentous cells (Fig. 4C, D). Transverse section through intergeniculum showing circle-ova shape with many lateral vacuoles and polygonal central cells (Fig. 4E).

During summer 2020, *C. officinalis* thallus appeared light red in color with 3.5–4.0 cm long and 1.0–1.2 cm of lateral branch (Fig. 5A). The number of terminal flattened intergenicula were three, sometimes more (Fig. 5B). The L. S. through mature intergeniculum showed symmetrical cells with few intercellular spaces and many peripheral tetrasporangial conceptacle (Fig. 5C). The geniculum cells were medullary in the core region and irregularly polygonal in the peripheral region (Fig. 5D, E). Cross-section showing peripheral tetrasporangial conceptacle during different stages and calcified peripheral cell layers (Fig. 5F).

As revealed by Fig. 6, *J. rubens* thallus exhibited reddish brown color during autumn 2019. The thallus was 1.0 cm length and 0.4 cm lateral branch of thin cylindrical appearance (Fig. 6A). The thallus was of regular bi-forked (dichotomous) branching with distinct geniculum regions and slender intergenicula (Fig. 6A). The L. S. showed intergenicula of spherical entwined medullary cells without large intercellular spaces (Fig. 6C). Arrow points to genicula forming at dichotomy. Transverse section of intergeniculum filament showed cortex with a single layer of epithelium cells in red (d) or without epithelium cells (Fig. 6E, F, respectively).

During winter 2019, the thallus of *J. rubens* was reddish orange in color, of 1.5–1.9 cm length and 0.5 cm lateral branch (Fig. 7A). The thallus was similar in structure to the autumn thallus with regular dichotomous branching and distinct geniculum regions (Fig. 7B). The L. S. of thallus showed intergeniculum with lateral enter fused medullary cells and genicula forming at dichotomy (Fig. 7C). The transverse section of intergeniculum filament showing cortex with a single layer of epithelium cells in blue (Fig. 7E).

During spring 2020, a similar thallus structure of *J. rubens* was observed. The thallus was brown–red in color; of 1.0–0.8 cm length and 0.5 cm lateral branch expansion with a forked dichotomous branching (Fig. 8A, B). The L. S. of thallus showed intergeniculum medullary cells and peripheral cortical cells with sporangial cavities (Fig. 8C). The genicula were formed at dichotomy of the filaments with calcified elongated cells. The arrow points to the lateral empty conceptacle (Fig. 8D). The transverse section of intergeniculum filament showing cortical cells with a single layer of epithelium cells (Fig. 8E).

During summer 2020, an unexpected vanishing of *J. rubens* was observed. Instead, *A. rigida* was recorded. Thallus showed densely tufted brown color that was growing on rocks. It was 1.6–2.0 cm in length with 0.4 cm lateral branch span (Fig. 9A). The thallus was dichotomously branched with short intergenicula; arrows point to protrusions of lateral and under-genicula conceptacles (Fig. 9B). L.S. of thallus showed genicula and intergenicula tiers (Fig. 9C). The genicula were formed at dichotomy with elongated calcified cells. The intergenicula showed symmetrical tiers of medullary cells without intercellular spaces (Fig. 9C). At the tips of the dichotomy under geniculum and/or at lateral sides of the intergenicula empty tetrasporangial conceptacle appeared; sometimes filled with spores (Fig. 9D, E). The transverse section of the intergeniculum filament showed tiers of cortical cells with a single layer of epithelium cells and tetrasporangial lateral conceptacles (Fig. 9F).

Collectively, it was noticed that these morphological and anatomical features were specific for each species and did not notably varied between seasons. This might be due to the ability of these species to acclimatize with the environmental changes among seasons. However, in the presented study, no sever ecosystem conditions e.g. hot temperature, desiccation, water acidification….etc. were recorded, which would stimulate significant changes in the morphological and anatomical features of the studied species. The main observed differences between the 3 species in their morphological and anatomical features were listed in Table 2. Many studies in the literature supported our findings and descripted similar morphological and anatomical results for the studied species such as Pardo et al. (2015); Cormaci et al. (2017), and Calderon et al. (2021).

**Table 2:** Morphological assessment of the studied species in the Eastern Harbor during 2019–2020

Description	*C. officinalis*	*J. rubens*	*A. rigida*
Color of Thallus	Red, red-pink	Reddish brown	Brown
Crustose base diameter (mm)	150–230	100- 150	150–300
Frond Branching	Trichotomous, dense and regular	Dichotomous, tufts and irregular	Dichotomous, dense and segmented
Gaps between successive lateral branches	Conspicuous	Inconspicuous	Conspicuous
Shape of intergenicula cells	Flatted-cylindrical	Spherical	Spherical
Length of intergenicula of main branches (cm)	2.0–4.0	1.0–2.0	1.6–2.0
Intergenicula of terminal branches (cm)	0.7–2.0	0.4–0.5	0.4
Intergenicula dimensions’ of main branches: length × diameter (μm)	3007–3954 × 424–436	988–2005 × 323–342	1587–2113 × 543–623
Genicula dimensions’ of main branches: length × diameter (μm)	55–80 × 235–277	38–45 × 145–168	65–78 × 397–413

### Molecular phylogenetic analysis of the collected calcareous species

**Fig. 10:**
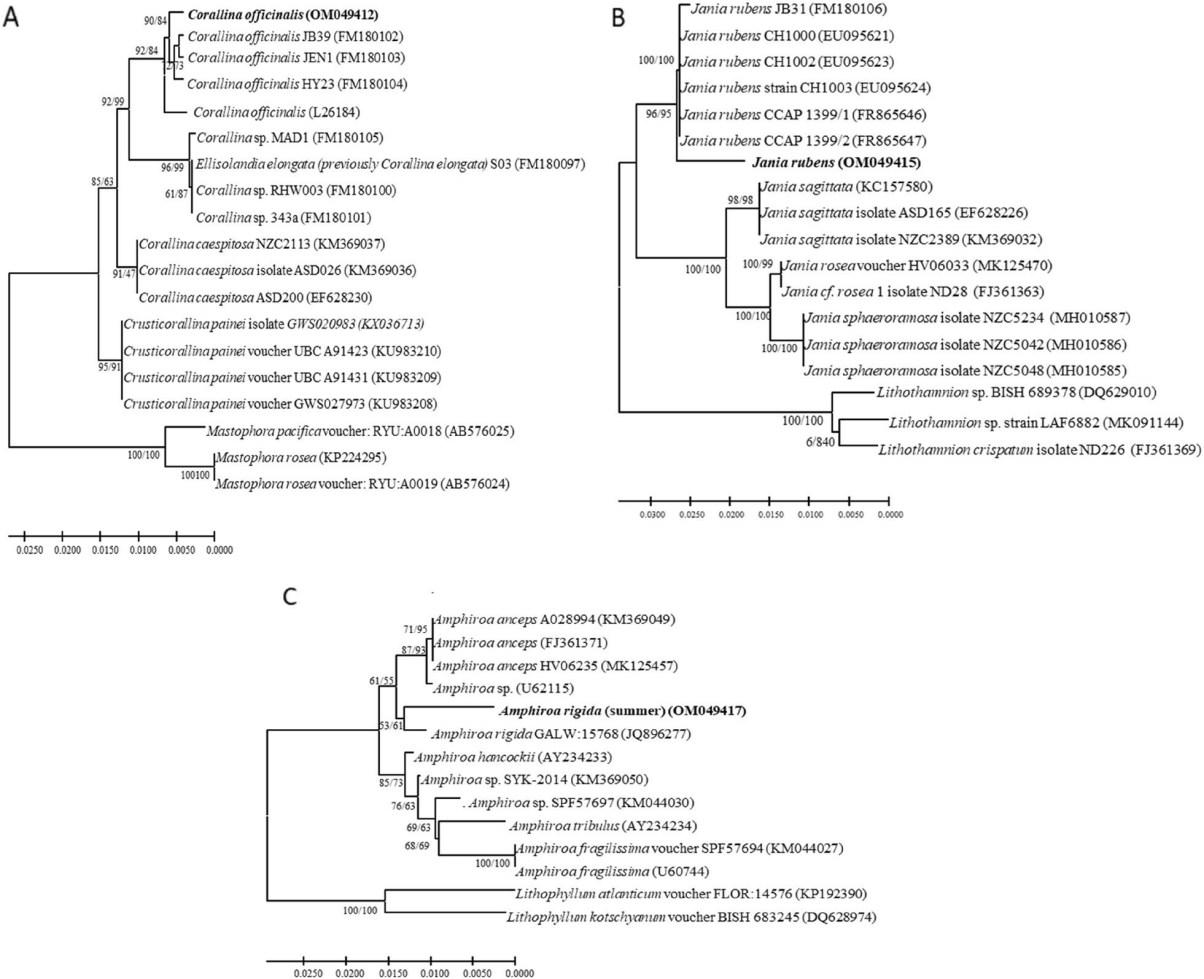
Maximum Likelihood (ML) dendrogram showing calcareous red species in different seasons based on 18S nucleotide sequences. Bootstrap values greater than 70 are shown on both trees in order of Maximum Likelihood bootstrap (left) and Maximum Parsimony (MP) (right). The identified species in this study were marked in bold. Distance scale was shown below each tree

The MP and ML phylogeny produced the same topology and comparable bootstrap support values of the three species. The sequences of the three seaweeds in different seasons have been submitted to Genbank. GenBank accession numbers of the identified species are shown in Fig. 10. Within the *Corallina* phylogram, the 18S rRNA gene phylogeny was inferred from 781 bp of the 17 algal strains and three strains as an out-group (Fig. 10A). The phylogeny analysis produced a MP tree with a tree length of 63 changes, the consistency index (CI) was 0.873, retention index (RI) was 0.952 and rescaled consistency index (RC) was 0.831 and had log likelihood of -1417.91 in (ML). The phylogeny showed 4 species of distinct clades of *C. officinalis, C. elongate, C. caespitosa* and *Crusticorallina painei*. The *Corallina* sample was grouped in one clade of *C. officinalis* with a high similarity of 99% with bootstrap support of 90/84 for ML and MP, respectively. The intraspecific variation of *C. officinalis* in different in *C. officinalis clade* was 0−6 bp.

Within *Jania* phylogram, the phylogeny analysis produced a MP tree with a tree length of 85 changes, the CI was 0.925, RI was 0.974 and RC was 0.902 and log-likelihood was -1505.6 in (ML). Two main clades were observed, the first clade of *J. rubens*, where the collected *Jania* were grouped with 98% similarity and 96/95 bootstrap support for, ML and MP, respectively. However, there was a low intraspecific variation of 0−18 bp within the clade of *J. rubens*. While the second clade is divided in to two sister clades of *J. sagittata* and 2^nd^ sister clade is separated to two sup-sister clades of *J. rosea* and *J. sphaeroramosa* (Fig. 10B).

On the other hand, the phylogeny analysis of *Amphiroa* produced a MP tree with a tree length of 88 changes, the CI was 0.807, RI was 0.882 and RC was 0.782 and had log likelihood of − 1620.06 in (ML). The summer collected *Amphiroa* has grouped in *A. rigida* clade with 98.61% similarity with the only 18S rRNA sequence found in the gene bank of *A. rigida* (JQ896277) and showed a bootstrap of 53/61 bootstrap support for ML and MP, respectively (Fig. 10C). This low bootstrap is due to the anonymous bp in the position 595–619 in the nucleotide sequence of *A. rigida* (JQ896277). The three species are set as mutually monophyletic, as shown in some other analyses (Broom et al. 2008; Smith et al. 2012).

### Seasonal variation in the phytochemicals content of the collected calcareous species

As shown in Table 3, a considerable variation in the phytochemicals (secondary metabolites) content was recorded between the gathered species at the season level. The winter season exhibited the plausible content of phytochemicals, and so were the summer and spring. At the phytochemical level, phenolic, terpenoids, and steroid compounds were dominant in all species during all seasons. Tannins, saponins and alkaloids components also obviously existed. Flavonoids, coumarins and quinones rarely occurred, while cardiac glycosides were never shown. Presence of these compounds enabled the studied species to better perform under cold temperature in winter and autumn seasons. These compounds also can act as antifouling, anti-grazing, and epiphytes deterrence (McCoy and Kamenos 2015), thus supporting the competitive interaction of these coralline species with other biota in their habitats. This may well relate with the abundance results of *Corallina* and *Jania* species around the year of the present study (Fig. 1). In this connection, Kumar et al. (2015) stated that screening secondary metabolites in different algae is an urgent process since these compounds play a critical role in their persistence capabilities in various habitats and as protective defense systems under harsh environmental conditions. Therefore, the present study suggests coralline seaweeds as a renewable source of bioactive compounds of many biological activities due to their richness in vital phytochemicals around the seasons of the year.

**Table 3:** Seasonal variation in the phytochemicals content of the methanolic extract of the studied calcareous species (+ means present, − means absent)

Season	Species	Alkaloid	Phenolic	Terpenoids	Tannins	Saponins	Steroid	Flavonoids	Coumarins	Quinones	Cardiac glycosides
Autumn 2019	*C. officinalis*	−	+ +	+	−	−	+	−	−	+	−
	*J. rubens*	+	+ +	+ +	+ +	−	+ +	+	+	−	−
Winter 2019	*C. officinalis*	+	+ +	+ +	−	+	+ +	+	+	+	−
	*J. rubens*	+	+ +	+ +	+ +	+	+ +	+	−	−	−
Spring 2020	*C. officinalis*	+	+ +	+	+	+	+	−	−	+	−
	*J. rubens*	−	+ +	+ +	+ +	+	+ +	**+ **	−	−	−
Summer 2020	*C. officinalis*	+	+ +	+	+ +	+	+	−	−	−	−
	*A. rigida*	−	+ +	+	−	+	+	−	−	+	−

### Seasonal variation in the biochemical profiling of the collected calcareous species

Figure 3 shows the proximate biochemical content (%) of the investigated calcified species in different collection seasons. These species generally favored the accumulation of carbohydrates followed by proteins rather than lipids (El-Sheekh et al 2021; El Zokm et al. 2020). For *C. officinalis*, proximate contents exhibited no great fluctuation, with the highest estimated record in winter (45.91, 40.33 and 17.13% DW) and in spring seasons (44.01, 40.83 and 13.87% DW) for carbohydrates, proteins, and lipid values, respectively. This is consistent with a previous study (Osman et al. 2020), who observed the highest carbohydrate content of the Mediterranean *C. officinalis* in winter. Regarding *J. rubens,* the same order of the biochemical contents were observed where the highest contents were recorded in winter (55.50, 27.94 and 12.53% DW) and in autumn (52.16, 33.36 and 11.30% DW), followed by the spring season (49.93, 22.70 and 11.62%) for carbohydrates, proteins, and lipid contents, respectively. For *A. rigida,* collected only in summer, the proximate values recorded for carbohydrates, proteins, and lipids were 43.24, 28.79, and 13.35% DW, respectively.

**Fig. 11:**
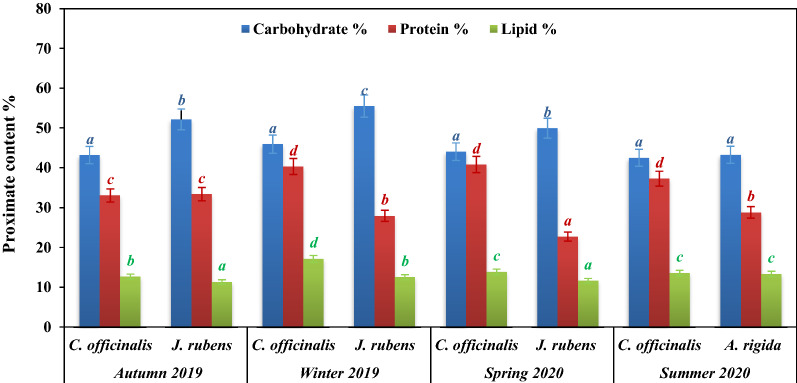
Proximate contents (%) of seasonally collected calcified species

Although accumulation of carbohydrates, proteins then lipids were the prevailing pattern for all collected species, the contents of each component showed a significant variation between the three species at *p* < 0.05 level of significance among studied seasons (Fig. 11). Higher contents of these compounds were recorded during winter and autumn seasons, especially with *J. rubens*. This indicates a greater photosynthetic capacity derived from the enlarged surface area of the lateral branches. In addition, accumulation of these proximate compounds evidenced for seasonal acclimation of the three species to differing levels of light irradiance, temperature, salinity and nutrients in summer and winter seasons during the study period.

### Seasonal variation in the pigment content of the collected calcareous species

Additional file 1: Figure S1 shows the content (as mg/g fresh weight) of chlorophyll a, total chlorophyll and carotenoid pigments of the studied species. In all seasons, total chlorophyll presented the highest values. *C. officinalis* showed the maximum quantities of these pigments in spring (75.23, 82.51 and 31.99 mg/g FW) followed by winter (68.86, 77.43 and 35.40 mg/g FW), while autumn season displayed the lowermost content (40.60, 46.76 and 12.79 mg/g FW) for chlorophyll *a*, total chlorophyll and carotenoid pigments, respectively. These results can be explained by the highest flourishing and biomass of *C. officinalis* that was recorded in winter (Osman et al. 2020). For *J. rubens* pigment contents, it represented 102.77, 127.73 and 32.97 mg/g FW in winter, followed by 93.02, 100.30 and 35.46 mg/g in autumn and finally, 86.31, 94.07 and 21.98 mg/g FW in spring for the same ordered pigments, respectively. In summer, *A. rigida* showed lower pigment contents of 56.94, 62.34, and 25.54 mg/g FW for chlorophyll *a*, total chlorophyll and carotenoid pigments, respectively.

Moreover, the content of phycobiliproteins pigments in the investigated calcareous species was displayed in Additional file 1: Figure S2 in relation to the collection seasons. The maximum phycobiliproteins content for *C. officinalis* was recorded in winter, followed by spring season then in autumn and finally in summer, so that they were following the same trend of chlorophylls and carotenoids. For *J. rubens,* phycobiliproteins content was the highest in autumn, followed by winter then spring. The significant decline in the pigments content during high temperature reflects a decrease in photosystem capacity, probably as a defense system to ameliorate the rise in temperature during the summer season. Similar observations were reported by Ismail and Osman (2016); Pereira et al. (2012).

Abiotic factors had a direct impact on the algal photosynthetic pigments. The temperature of water plays a critical role with respect to the physiological process of the algae. As irradiance and temperature decreased in autumn, chlorophyll *a* and phycobiliprotein levels raised and remained high throughout winter, typically reaching maximum levels in winter (Guihéneuf et al., 2018). This results were confirmed by Pearson correlation analysis which showed a negative correlation between the recorded temperature values and the carotenoids and phycocyanin pigments of the tested species along seasons (Table 4). This negative correlation can be explained by at low irradiance and temperature pigment concentrations increased to maintain the photosynthetic rate (Guihéneuf et al., 2018). This implies that temperature did not affect the metabolism and the photosynthetic pathways of these species, besides confirming their ability to tolerate ambient temperate circumstances. However, some other studies have reported the susceptibility of red calcareous seaweed species to the rising temperature (Basso 2012; Kim et al. 2018). In addition, as shown in Table 4, the positive effect of salinity was observed on the pigment apparatus of phycobiliproteins and carotenoids in the studied calcareous species but negatively correlated with the lipids content. This reflected that salinity conditions during this study were suitable for the growth and metabolism of the tested coralline species. Similar effective influence was noticed for the nitrites (NO_2_) content of the ambient seawater in relation to the proximate and pigment composition of the three species.

**Table 4:** Correlation coefficient values (*r*) between seawater physicochemical parameters and the biochemical compositions of the studied calcareous species*

Species parameters		Temperature	Salinity	PH	DO	NO_3_	NO_2_	NH_4_	PO_4_
Abundance		0.049	− 0.037	**0.469**	**0.427**	− 0.526	− 0.348	**0.718**	**− 0.486**
Carbohydrates		0.054	− 0.155	0.135	0.022	0.110	− 0.294	− 0.331	− 0.072
Lipids		0.272	** − 0.543**	0.062	− 0.070	** − 0.461**	** − 0.556**	0.121	** − 0.540**
Proteins		0.182	− 0.097	− 0.237	− 0.222	− 0.158	− 0.008	0.058	− 0.113
Chlorophyll a		− 0.177	0.115	0.192	0.255	− 0.068	0.152	**0.456**	0.040
Total chlorophyll		− 0.250	0.174	**0.454**	**0.437**	− 0.054	0.213	**0.457**	0.082
Carotenoids		** − 0.604**	**0.466**	**0.554**	**0.618**	**0.456**	**0.427**	0.080	**0.465**
PC		** − 0.455**	**0.522**	0.021	0.239	0.087	**0.673**	**0.502**	**0.411**
APC		− 0.083	**0.544**	− 0.374	− 0.179	− 0.043	**0.572**	**0.389**	0.244
PE		− 0.232	**0.456**	− 0.210	− 0.008	0.055	**0.624**	**0.452**	**0.438**

^*^Bold values are significantly correlation at *P* < 0.05 level

Meanwhile, for the pH and proximate contents relationship, the obtained results show no correlation linking these coralline species, except for total chlorophyll and carotenoid pigments content. Coralline species have a high response to marine acidification (Peña et al. 2021; Cornwall et al. 2022), which was not observed in the current study; since the pH value was tangled in the light, alkaline range (Table 1). The same observation was noticed for dissolved oxygen and nitrates content. Also, ammonium content in the water was positively correlated to chlorophyll a, total chlorophyll and phycobiliproteins contents of the studied species. A negative correlation was detected between PO_4_, NO_3_ and NO_2_ contents of the seawater in relation to lipids accumulation in the studied species. The positive correlation between the nitrogenous nutrients of seawater and the all pigment (phycobiliproteins, carotenoids and chlorophyll a) contents of the studied seaweeds rather than with their proximate (carbohydrate, protein and lipid) contents can be explained on the basis that the seaweeds firstly need these nutrients to motivate various metabolic pathways; especially the photosynthetic process (Boundir et al., 2019). This will, eventually, lead to the synthesis and accumulation (or storage) of various organic proximate products. These results agreed with Marinho-Soriano (2012), who demonstrated the important role of nitrogenous nutrients in pigment biosynthesis. And also with Xu et al. (2020) who stated the sufficiency of nitrogenous compounds for the production of chlorophyll and phycobiliproteins as protective substances.

Moreover, there was a detected positive correlation between seawater phosphorous (PO_4_) content and carotenoids, phycocyanin, and phycoerythrin pigments content of the studied species where PO_4_ had a vital role in energy transfer mediated by ATP and other high energy compounds existing in the photosynthesis and respiration process (Johansson 2002). Collectively, from the results in Table 4, it seems that carotenoids and phycobiliproteins were the major parameters that related to the change in the environmental seawater, which points out their main role in the physiology of these seaweeds, including carbohydrate anabolism. In this connection, it has been reported that red algae are almost entirely marine found deep in the water column, especially for coralline species which were found up to 268 m below the water surface (İlknur and Yücesan 2012). Due to this, red algal species cannot only rely on chlorophylls to absorb light at the ultraviolet region of the spectrum. Instead, they use other accessory compounds, mainly carotenoids and phycobiliproteins to get light from different spectrums. Carotenoids can absorb light in the blue range spectrum while, at the same time, preventing oxidative stress on chlorophyll molecules due to the photodamage effect. Likewise, phycobilins can absorb light in the red, orange, yellow, and green wavelengths of the spectrum that are not well absorbed by chlorophyll a, thus, enables the photosynthetic process to proceed efficiently in these coralline species (İrkin and Erduğan 2014; Ismail and Osman 2016).

### Calcium carbonate, elements contents of coralline samples

The CaCO_3_ content in the tested species showed variation between species and seasons (Table 5). The concentration was the highest during winter for *C. officinalis* (2.247 ± 0.37 mg/g). Generally, *J. rubens* accumulated lower contents of CaCO_3_ than *C. officinalis,* with the highest record of 1.488 ± 0.09 mg/g during autumn season. This variation may be related to the environmental parameters and the algal development stage (de Carvalho et al. 2022; Teichert and Freiwald 2014).

**Table 5:** Elements and calcium carbonate content of the studied calcareous species*

Element	Cu	Cd	Cr	Mn	Ni	Pb	Zn	Na	Ca	K	Mg	Fe	CaCO_3_
Season	µg/g							mg/g					
*C. officinalis* (winter)	0.043 ± 0.01	1.469 ± 0.6	0.004 ± 0.0	85.066 ± 1.32	0.004 ± 0.0	32.186 ± 0.75	35.392 ± 1.15	4.191 ± 0.21	12.352 ± 0.12	21.154 ± 1.04	16.335 ± 0.62	0.695 ± 0.08	2.247^**a**^ ± 0.41
*C. officinalis* (autumn)	0.064 ± 0.01	5.530 ± 0.11	0.009 ± 0.0	108.289 ± 1.22	0.009 ± 0.0	25.749 ± 0.45	49.096 ± 1.75	5.827 ± 0.32	14.704 ± 0.14	16.923 ± 1.22	22.715 ± 0.43	0.591 ± 0.03	1.725^**b**^ ± 0.32
*C. officinalis* (summer)	0.028 ± 0.001	3.112 ± 0.31	0.003 ± 0.0	58.164 ± 1.02	0.003 ± 0.0	9.170 ± 0.32	4.962 ± 0.55	2.865 ± 0.74	11.499 ± 0.11	6.027 ± 0.84	11.169 ± 0.71	0.560 ± 0.01	1.617^**b**^ ± 0.25
*C. officinalis* (spring)	0.147 ± 0.02	1.196 ± 0.02	0.002 ± 0.0	50.776 ± 0.82	0.2880.01	22.478 ± 0.30	25.896 ± 0.45	2.501 ± 0.41	22.530 ± 0.08	14.774 ± 0.78	9.751 ± 0.94	0.615 ± 0.03	1.642^**b**^ ± 0.15
*J. ruben* (winter)	0.054 ± 0.02	4.076 ± 0.31	0.005 ± 0.0	56.525 ± 0.62	0.005 ± 0.0	21.183 ± 0.26	40.983 ± 0.85	1.648 ± 0.52	12.919 ± 0.12	13.923 ± 1.80	10.855 ± 0.32	0.988 ± 0.05	1.311^c^ ± 0.22
*J. ruben* (autumn)	0.0160.002	0.111 ± 0.002	0.002 ± 0.0	25.878 ± 0.12	0.002 ± 0.0	9.926 ± 0.12	22.720 ± 0.45	0.755 ± 0.33	10.860 ± 0.07	6.524 ± 0.32	4.970 ± 0.94	0.686 ± 0.06	1.488^**b**^ ± 0.09
*J. ruben* (summer)	0.200 ± 0.05	5.005 ± 0.31	0.020 ± 0.0	113.514 ± 0.94	0.020 ± 0.0	6.607 ± 0.09	94.535 ± 1.25	3.310 ± 0.21	20.898 ± 0.16	4.342 ± 0.56	21.798 ± 0.45	2.874 ± 0.08	1.297^**c**^ ± 0.32
*Amphiroa rigida* (spring)	0.0)64 ± 0.01	3.707 ± 0.21	0.006 ± 0.0	15.211 ± 0.11	0.006 ± 0.0	6.072 ± 0.05	56.447 ± 0.95	5.940 ± 0.18	13.479 ± 0.10	33.991 ± 1.64	2.921 ± 0.21	0.669 ± 0.04	1.120^**c**^ ± 0.17

^*^Values are means of three replicates ± SD

Results in Table 5 also showed the elements content of the investigated red species. *C. officinalis* showed maximum contents of Mn, Pb, Zn, Ca, Mg, K, Na, and Cd during winter and autumn seasons. In spring, *C. officinalis* displayed notable contents of Ca, Pb, Ni, and Cu. On the other hand, *J. rubens* showed the highest accumulation activity during the summer season for Mn, Zn, Mg, Ca, Cd, Fe, and Cu, while presenting high Zn and Pb contents during winter. Besides, *A. rigida,* which occurred only in spring season, showed maximum contents of Zn, K, and Na. According to these results, the accumulation of these elements depended on the species, the collection season, and the estimated element. These results were in conformity with Dango et al. (2015) and Kim et al., (2018).

**Fig. 12:**
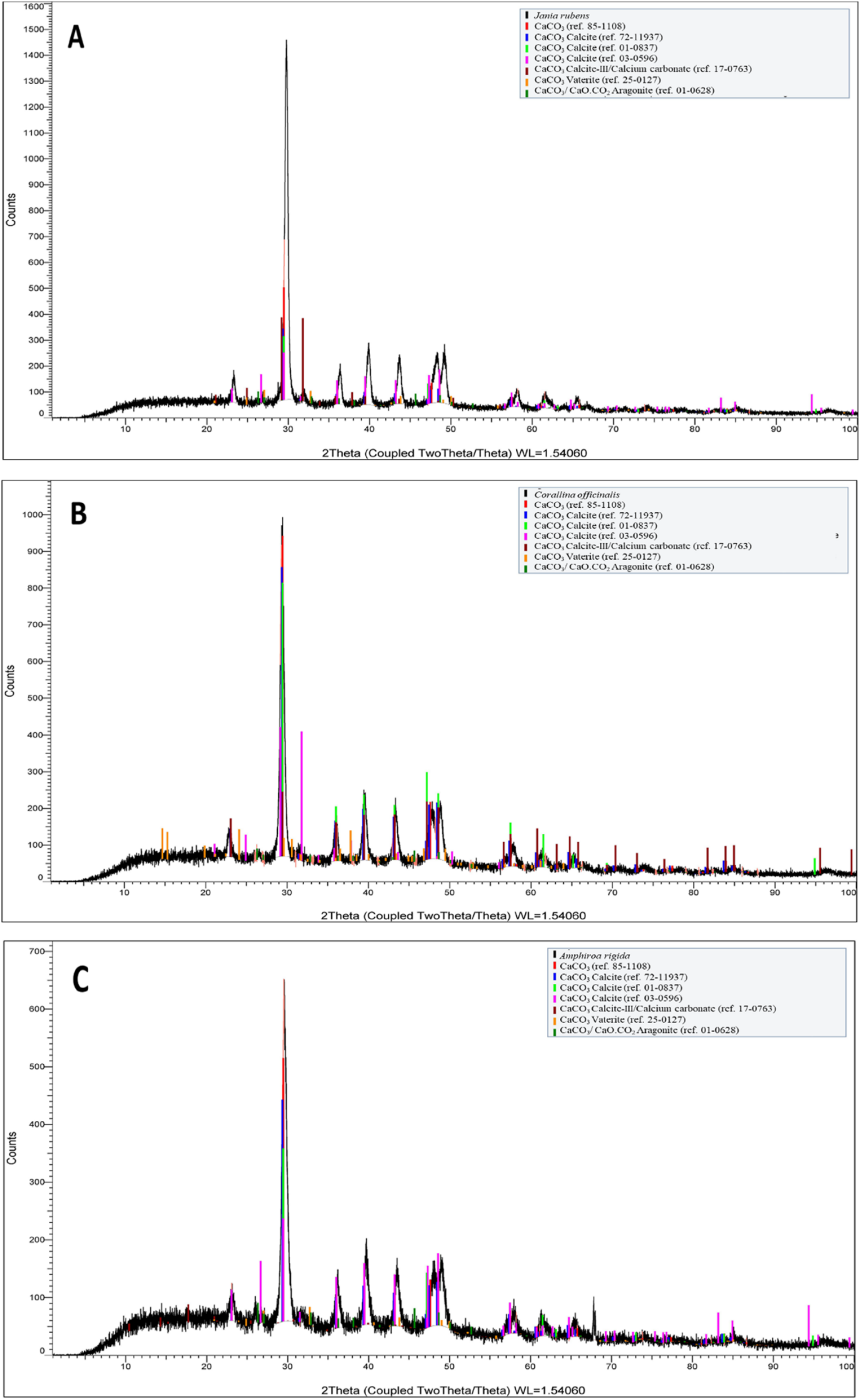
X-ray diffraction patterns of the selected seaweed: **A** *Jania rubens* and **B** *Corallina officinalis*; **C** *Amphiroa rigida*

X-ray diffraction analysis was conducted to distinguish between the three coral species' crystal structural compositions. The XRD confirms that CaCO_3_ (≤ 16.89%) is the main salt in the tested species that was present in different ratios and polymorphic forms (Additional file 1: Table S1 and Fig. 12). In general, calcite (≤ 39.45%) is the main mineral that is deposited in red algae, however, corallinacean thalli can create various inorganic crystal deposits of calcium or magnesium calcite (Bianco-Stein et al. 2021). Increased temperatures have been linked to a decrease in net calcification, growth, and reproduction of calcified species, according to Basso (2012). Besides calcite, a fraction of aragonite was also present in a small ratio range of 1.61–4.33%. Aragonite is a modified type of CaCO_3_ depositions, which is exclusive mineral type of a given genus or family Randi et al. (2021). In addition to vaterite crystals, other types of less stable CaCO_3_ depositions were detected as hexagonal form in *A. rigida* and *J. rubens* and as orthorhombic form in *A. rigida* only. Furthermore, calcite-III I calcium carbonate, which are intermediary between calcite I and aragonite crystals, was observed in *J. rubens* (30.31%) and *C. officinalis* (18.67%) in orthorhombic and rhombo H. axes forms, respectively. However, *C. officinalis* contained only monoclinic crystal system as calcium oxalate (4.41%). The mineral content of these corallinacean species varied with their growth rate, age and environmental parameters (Mohammadi 2020). These variations in polymorphs of CaCo_3_ depositions may precipitate in peculiar situations largely for kinetic reasons in the tested species (Firdous et al. 2021).

## Conclusion

The abundance and diversity of the Rhodophyta species are generally affected by the physicochemical parameters of the surrounding seawater of their habitats. The present study recorded lower diversity of the red coralline species than recorded in the past years for the same location. During seasons of 2019−2020, only three species of red calcified algae were recorded viz*. Corallina officinalis, Jania rubens,* and *Amphiroa rigida.* The 18S rRNA confirmed the identified species and showed low intraspecific variation within the species. A positive correlation between salinity level, the nitrogenous nutrients of the seawater, and the pigment contents (phycobiliproteins, carotenoids and chlorophyll a) of the studied seaweeds was recognized. Carotenoids and phycobiliproteins were the major parameters that related to the change in the environmental seawater criteria, which affect the physiology of these seaweeds, including carbohydrate anabolism. In addition, the biochemical contents, elemental composition, and calcification capability of these species were elevated in winter and lowered in summer; and were dependent on the species, the collection season, and the estimated element. Calcium carbonate as calcite-III I were deposited in significant content in *J. rubens* and C. officinalis in orthorhombic and rhombo H. axes forms.

It should be mentioned that the change in physicochemical parameters of the seawater was not the common reason for this effect since they exhibited no major variation along seasons. Alternatively, global climate changes such as rising temperature and sea level rise might also interfere coralline algae distribution. In the study area of Eastern Harbor, stronger tidal waves strike the shores affecting their infrastructure, and consequently, enforce intensive shore line hardening practices. In this connection, more future studies should be conducted to assess the possible usage of these coralline species and other macroalgae species as biomarkers for climate changes and anthropogenic effects.

## Supplementary Information

**Additional file 1: Figure S1.** Chlorophyll a, total chlorophyll and carotenoid pigments content (mg/g fresh weight) of seasonally tested calcified species. **Figure S2. **Phycocyanin (PC), allophycocyanin (APC) and phycoerythrin (PE) pigments content (mg/g FW) of seasonally collected calcified species. **Table S1.** Characterization of the XRD patterns of the studied calcareous species (a, b and c indicate dimensions of the crystal arms)
