# Supplementary material for: Morpho-anatomical, and chemical characterization of some calcareous Mediterranean red algae species
Source: Bot Stud. 2023 Apr 18;64:10. doi: 10.1186/s40529-023-00373-0 (PMC10113420; doi:10.1186/s40529-023-00373-0)
Supplement: Supplementary file 1 — Additional file 1: Figure S1. Chlorophyll a, total chlorophyll and carotenoid pigments content (mg/g fresh weight) of seasonally tested calcified species. Figure S2. Phycocyanin (PC), allophycocyanin (APC) and phycoerythrin (PE) pigments content (mg/g FW) of seasonally collected calcified species. Table S1. Characterization of the XRD patterns of the studied calcareous species (a, b and c indicate dimensions of the crystal arms) [file 40529_2023_373_MOESM1_ESM.docx]

**Figure 2s.** Phycocyanin (PC), allophycocyanin (APC) and phycoerythrin (PE) pigments content (mg/g FW) of seasonally collected calcified species

**Table 1s.** Characterization of the XRD patterns of the studied calcareous species (a, b and c indicate dimensions of the crystal arms)

|  | Compound Name | Formula | Crystal System | Content  % | Pattern (Tune Cell) | **a** | **b** | **c** |
| --- | --- | --- | --- | --- | --- | --- | --- | --- |
| ***Jaina rubens*** | Calcium Carbonate | CaCO_3_ | Rhombo .H. axes | 12.11 | ref. 85-1108 | 4.980 |  | 17.019 |
|  | Calcite | CaCO_3_ | Rhombo. H. axes | 8.08 | ref. 72-1937 | 4.994 |  | 17.081 |
|  | Calcite | CaCO_3_ | Rhombo. H axes | 23.26 | ref. 01-0837 | 4.983 |  | 17.019 |
|  | Calcite | CaCO_3_ | Rhombo. H. axes | 17.28 | ref. 03-0596 | 4.983 |  | 17.020 |
|  | Calcite-III I | CaCO_3_ | Orthorhombic | 30.31 | ref. 17-0763 | 8.900 | 8.420 | 7.140 |
|  | Vaterite | CaCO_3_ | Hexagonal | 4.75 | ref. 25-0127 | 7.150 |  | 16.940 |
|  | Aragonite | CaCO_3_/ CaOCO_2_ | Orthorhombic | 4.21 | ref. 01-0628 | 4.940 | 7.940 | 5.720 |
| ***Corallina fficinalis*** | Calcium Carbonate | CaCO_3_ | Rhombo. H. axes | 13.63 | ref. 85-1108 | 4.980 |  | 17.019 |
|  | Calcite | CaCO_3_ | Rhombo. H. axes | 12.91 | ref. 72-1937 | 4.994 |  | 17.081 |
|  | Calcite | CaCO_3_ | Rhombo. H. axes | 39.45 | ref. 01-0837 | 4.983 |  | 17.019 |
|  | Calcite | CaCO_3_ | Orthorhombic | 9.31 | ref. 17-0763 | 8.900 | 8.420 | 7.140 |
|  | Calcite-III I | CaCO_3_ | Rhombo. H. axes | 18.67 | ref. 02-0629 | 4.983 |  | 17.020 |
|  | Calcium oxalate | C_2_CaO_4_/CaC_2_O_4_ | Monoclinic | 4.41 | ref. 21-0838 | 9.794 | 14.745 | 6.306 |
|  | Aragonite | Ca CO_3_/ CaOCO_2_ | Orthorhombic | 1.61 | ref. 01-0628 | 4.940 | 7.940 | 5.720 |
| ***Amphiroa rigida*** | Calcium Carbonate | CaCO_3_ | Rhombo. H. axes | 16.89 | ref. 85-1108 | 4.980 |  | 17.019 |
|  | Calcite | CaCO_3_ | Rhombo. H. axes | 14.93 | ref. 72-1937 | 4.994 |  | 17.081 |
|  | Calcite | CaCO_3_ | Rhombo. H. axes | 37.59 | ref. 01-0837 | 4.983 |  | 17.019 |
|  | Calcite | CaCO_3_ | Rhombo. H. axes | 22.35 | ref. 03-0596 | 4.983 |  | 17.020 |
|  | Vaterite | CaCO_3_ | Hexagonal | 0.49 | ref. 72-1616 | 7.148 |  | 16.949 |
|  | Vaterite | CaCO_3_ | Orthorhombic | 3.41 | ref. 74-1867 | 4.130 | 7.150 | 8.480 |
|  | Aragonite | CaCO_3_/ CaOCO_2_ | Orthorhombic | 4.33 | ref. 01-0628 | 4.940 | 7.940 | 5.720 |
